# A protocol for noninvasive quantification of dietary fat absorption in mice

**DOI:** 10.1016/j.xpro.2026.104658

**Published:** 2026-06-25

**Authors:** Alvin P. Chan, Kelsey E. Jarrett, Rochelle W. Lai, Bethan L. Clifford, Kevin J. Williams, Elizabeth J. Tarling, Thomas Q. de Aguiar Vallim

**Affiliations:** 1 Division of Cardiology, Department of Medicine, David Geffen School of Medicine, University of California, Los Angeles (UCLA), Los Angeles, CA 90095, USA; 2 Division of Gastroenterology, Hepatology and Nutrition, Department of Pediatrics, Mattel Children’s Hospital, David Geffen School of Medicine, University of California, Los Angeles (UCLA), Los Angeles, CA 90095, USA; 3 Division of Endocrinology, Department of Medicine, David Geffen School of Medicine, University of California, Los Angeles (UCLA), Los Angeles, CA 90095, USA; 4 UCLA Lipidomics Laboratory, University of California, Los Angeles (UCLA), Los Angeles, CA 90095, USA; 5 Jonsson Comprehensive Cancer Center (JCCC), University of California, Los Angeles (UCLA), Los Angeles, CA 90095, USA; 6 Molecular Biology Institute, University of California, Los Angeles (UCLA), Los Angeles, CA 90095, USA; 7 Department of Biological Chemistry, David Geffen School of Medicine, University of California, Los Angeles (UCLA), Los Angeles, CA 90095, USA

**Keywords:** Cell Biology, Metabolism, Molecular Biology

## Abstract

Dietary fat absorption is a key determinant of energy balance, yet standard techniques to measure fat absorption are often invasive or challenging. Here, we present a protocol to non-invasively quantify intestinal fat absorption in mice during physiological feeding. We describe steps for preparing a diet containing a nonabsorbable lipid tracer and calculating fat absorption from fecal samples using gas chromatography-mass spectrometry (GC-MS), alongside complementary approaches for measuring fecal energy loss by oxygen bomb calorimetry and fecal fatty acid composition by GC-MS.

For complete details on the use and execution of this protocol, please refer to Chan et al.^1^

## Highlights

• Protocol for measuring dietary fat excretion and absorption in mice
• Instructions for preparing diets containing an indigestible lipid tracer
• Procedures for processing feces for bomb calorimetry and mass spectrometry

## Abstract (editor highlights)

Publisher’s note: Undertaking any experimental protocol requires adherence to local institutional guidelines for laboratory safety and ethics.

## Graphical abstract

**Figure:**
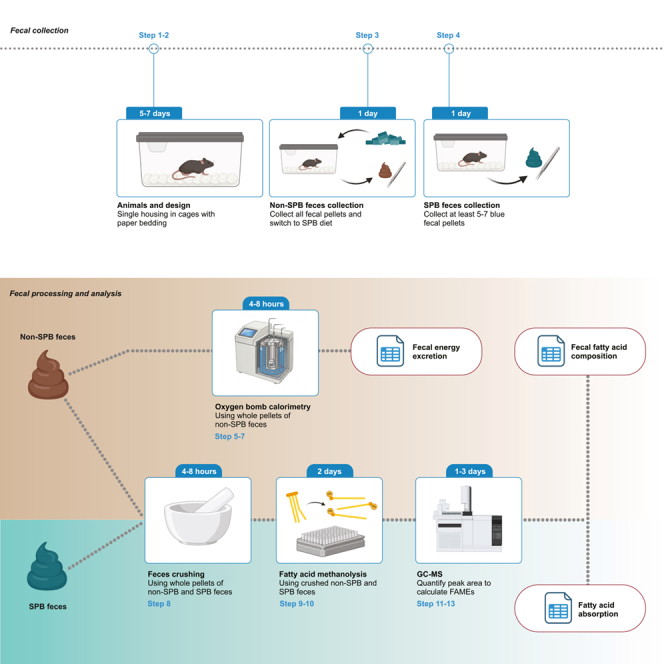

## Before you begin

Accurate quantification of dietary fat absorption is critical for studies investigating energy balance and metabolic disease, where changes in absorption can meaningfully contribute to overall energy homeostasis. When designing such studies, it is important to first select the mode of dietary fat absorption measurement that best supports your experimental aim. Our protocol provides detailed instructions for quantifying intestinal dietary fat absorption in mice across experimental paradigms during normal feeding, making it well suited for studies that aim to also investigate gut hormone signaling or bile acid metabolism.

Jandacek and colleagues originally developed a non-invasive approach using the indigestible lipid tracer sucrose polybehenate (SPB) to calculate percent dietary fat absorption from the ratios of behenic acid (C22:0) to other fatty acids in diet and feces. SPB closely resembles the physical properties of triglycerides but consists predominantly of behenic acid esterified to a sucrose backbone, rendering it resistant to hydrolysis.^2^ When incorporated into the diet, SPB passes unabsorbed into the feces and serves as an internal standard for quantifying dietary fatty acid absorption. Because endogenous behenic acid is present at only negligible amounts in the diet, it can be readily distinguished from other dietary fatty acids.

Established gavage-based radioactive^3^ or fluorescent tracer methods^4^ to measure intestinal fat absorption remain valuable depending on the research question and experimental context.^5^ These methods allow delivery of a standardized lipid dose for direct assessment of tissue uptake at defined endpoints but often require prolonged fasting and an acute lipid bolus that disrupts feeding patterns, gut hormone secretion, and bile acid physiology. Mesenteric lymph cannulation provides direct quantification of intestinal lipid uptake and chylomicron secretion but requires specialized surgical expertise and terminal endpoints.^6^ Because both approaches standardize lipid delivery, they do not account for the variability in spontaneous *ad libitum* food intake. In contrast, conventional fat balance studies, which consider food intake, rely on comprehensive measurement of food and feces over multiple days, making them labor-intensive and susceptible to collection errors.

Here we describe a protocol that has been optimized to quantify dietary fat absorption at weekly intervals in mice fed a Western diet to induce obesity. This protocol can be adapted for use in other animal models and diets, although optimization of the nonabsorbable lipid tracer concentration and adjustment of fecal collection time points may be required.

### Innovation

Building on the original work by Jandacek and colleagues,^2^ we introduce key optimizations that enhance physiological relevance and adaptability for complementary absorption measurements. First, we reduce the concentration and duration of SPB exposure, minimizing its potential impact on feeding and absorption. Second, we correct the fat absorption calculations for SPB impurity by accounting for the minor amounts of other fatty acid species present in SPB, improving quantification accuracy. Third, we incorporate a non-absorbable food dye to confirm passage of SPB into the feces for analysis. Lastly, we combine complementary analyses of fecal energy loss and fecal fatty acid composition using oxygen bomb calorimetry and GC-MS, respectively.

### Institutional permissions

All procedures in this protocol were performed in accordance with the Institutional Animal Care and Use Committee (IACUC) at the University of California, Los Angeles. Readers will need to acquire the necessary approvals from relevant institutional committees before commencing their research protocol.

### Prepare tools and supplies

1. A sufficient number of mice based on sample size calculations of the study protocol. We used 8-10 mice per group in most studies in Chan *et al*,^1^ though the number of mice may vary depending on the magnitude of the effect or biological variability.
2. A sufficient amount of experimental diet for the duration of the study.
3. Equipment, supplies, and chemicals for preparation of custom diet containing sucrose polybehenate (SPB):
  a. Weighing scale.
  b. Experimental diet.
  c. Food processor.
  d. SPB, store at 25°C in a tightly sealed container to avoid excessive heat and moisture.
  e. Blue food dye.
  f. Spatula.
  g. Pellet press.
  h. Airtight container.
  i. Refrigerator.
4. Supplies for feces collection:
  a. Paper cage bedding.
  b. Tweezers.
  c. Conical tube, 10 mL.
  d. Eppendorf tube.
  e. Refrigerator.
5. Equipment and supplies for fecal energy analysis by oxygen bomb calorimetry:
  a. Weighing scale.
  b. Oxygen bomb calorimeter.
  c. Oxygen gas cylinder.
6. Equipment, supplies, and chemicals for fatty acid analysis by gas chromatography-mass spectrometry:
  a. Liquid nitrogen.
  b. Hexane.
  c. Glass beakers.
  d. Glass syringes, 10 μL 25 μL, and 100 μL volumes.
  e. Glass tubes, 12 mL volume.
  f. Amber vial, 1.5 mL volume.
  g. GLC-96C reference fatty acid standard mix.
  h. 96-deep well rack system.
    i. 96-well aluminum plate base.
    ii. Glass flat vial inserts, 2.5 mL volume.
    iii. Molded PTFE lined silicone liner.
    iv. Aluminum cover with screw holes.
    v. Screws for aluminum cover, 2.5 mL.
  i. Glass graduated cylinder.
  j. Glass pipette, 1 mL, 10 mL, and 25 mL volumes.
  k. Electronic pipette.
  l. Trinonadecanoin (C17:1) internal standard.
  m. Methanol.
  n. Hydrochloric acid.
  o. Toluene.
  p. Pasteur pipette, or automated liquid handler (optional).
  q. Incubator.
  r. Sodium chloride.
  s. Centrifuge with a swing-bucket plate rotor.
  t. Glass flat vial inserts, 1.5 mL volume (for 96-well system using same aluminum plate base as above).
  u. Pierceable cap mats.
  v. Gas chromatography-mass spectrometer.

### Preparation of diet containing sucrose polybehenate

**Timing: 4 h**

This section describes preparation of an SPB-containing diet required for measurement of intestinal fatty acid absorption.

***Note:*** Allocate sufficient experimental diet to prepare the custom SPB-containing diet. Because the SPB diet is administered for 24 h and adult mice may consume up to ∼5 g/day, we recommend allocating ∼10 g of diet per mouse per single time point to account for variability and spillage. For this example, 200 g of diet is prepared for analysis of a single timepoint for 20 mice.

**CRITICAL:** The experimental diet should contain only negligible amounts of endogenous C22:0. If an appreciable amount exists in the diet, this can falsely lower the calculated fatty acid absorption in downstream analyses. Refer to troubleshooting – problem 1.

**Figure 1:**
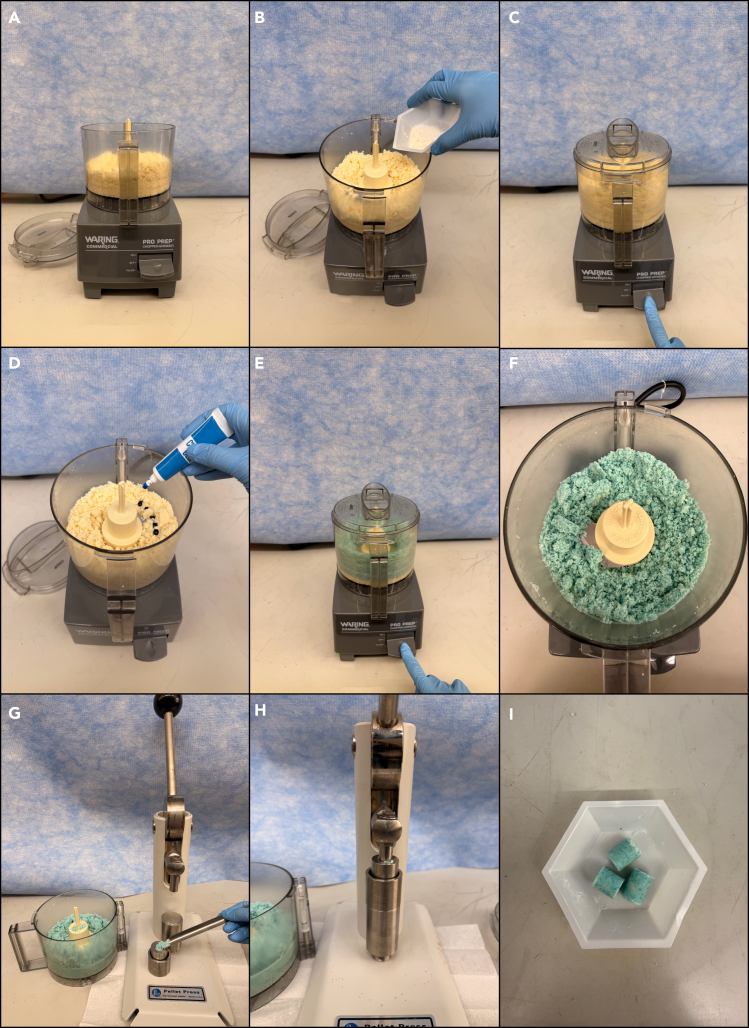
Stepwise preparation of Sucrose Polybehenate (SPB)-containing diet (A) Experimental diet is ground into a fine powder using food processor. (B and C) SPB is mixed into the powdered diet. (D–F) Blue dye is mixed into the SPB-containing powdered diet. (G–I) Powdered diet is reformed into pellets using pellet press.

***Note:*** We used a WCG75 food processor with 0.75 qt capacity, 3/4 horsepower (Waring Commercial, McConnellsburg, PA).

7. Using a food processor, grind 200 g of experimental diet into a fine powder. Perform grinding in small batches to ensure consistent and uniform results (Figure 1A).

8. Weigh the appropriate amount of SPB to achieve a final concentration of 1.5% (weight/weight). For this example, use 3 g SPB for the 200 g diet.

***Note:*** Set aside 10 mg of SPB into a separate container and reserve for gas chromatography-mass spectrometry (GC-MS) preparation later in Step 8e to determine the exact fatty acid composition of the SPB, which is required to calculate the percent absorption.

9. Add the SPB to the powdered diet in the food processor and grind until fully mixed (Figures 1B and 1C).
10. Add several drops of blue food dye to the SPB-containing powdered diet and grind until the blue color is achieved and evenly distributed throughout the mixture (Figures 1D–1F).

***Note:*** In our preparation, 5 drops were used for a 200 g batch, but the exact number of drops may vary depending on dye and batch size.

***Note:*** Although other color dyes can be used, blue is preferred because it allows easy assessment of uniform mixing and is readily visible against the white bedding during feces collection in later steps. Red dye should be avoided to prevent confusion with blood.

11. Form the SPB-containing diet into pellets using a pellet press according to the manufacturer’s instructions (Figures 1G–1I).

***Note:*** We used a 2811 pellet press (Parr Instrument Company, Moline, IL).

12. Store the SPB diet at 4°C in an airtight container until use. This diet can be stored for up to 6 months.

## Key resources table

**Table:** 

REAGENT or RESOURCE	SOURCE	IDENTIFIER
**Chemicals, peptides, and recombinant proteins**		

Triheptadecenoin (C17:1)	Nu-Chek Prep	Cat# T-404; http://www.nu-chekprep.com/
GLC96C lipid standards (mixed lipid standards)	Nu-Chek Prep	Cat# GLC96C; http://www.nu-chekprep.com/
Sucrose polybehenate	CarboMer, Inc.	Cas: 56449-50-4
Sodium chloride	Thermo Fisher Scientific	Cat# 640500

**Experimental models: Mice**		

Mice: C57BL/6J, male, 8-weeks-old	Jackson Laboratories	#000664

**Software and algorithms**		

Graphpad Prism V10	Graphpad	https://www.graphpad.com/
R	R Core Team	https://www.r-project.org
Microsoft Excel	Microsoft	N/A

**Other**		

Western diet	Research Diets	Cat# D12079B
100 μL, Microliter syringe, cemented needle	Hamilton Company	Cat# 80665
25 μL, Microliter syringe, cemented needle	Hamilton Company	Cat# 80400
10 μL, Microliter syringe, cemented needle	Hamilton Company	Cat# 80339
Glass flat vial inserts, 1.5 mL volume	Chrom Tech, Inc.	Cat# 96I-04150F-B
Glass flat vial inserts, 2.5 mL volume	Chrom Tech, Inc.	Cat# 96I-04250F-B
96-well plate base	Chrom Tech, Inc.	Cat# 96AL-12
Aluminum cover with screw holes	Chrom Tech, Inc.	Cat# 96-25SC
Screws for 2.5 mL Alum MTP	Chrom Tech, Inc.	Cat# 96AL-10
Liner, molded (96), PTFE/Sil, replacement liner	Chrom Tech, Inc.	Cat# 96M-16
Pierceable cap mats for 1 mL round	Chrom Tech, Inc.	Cat# 96-0566AS
7890B/5977A GC-MS	Agilent Technologies	N/A
DB-WAX UI column	Agilent Technologies	Cat# 122-7032 UI
6400 Isoperibol calorimeter with an 1138 oxygen bomb	Parr Instrument Company	N/A
2811 Pellet press	Parr Instrument Company	N/A
WCG75 Food processor	Waring Commercial	Cat# 6FTJ8

## Materials and equipment

**Table:** Acid methanolysis mix (for a 96-well plate)

Reagent	Amount	Notes
Methanol	106.4 mL	Use a glass pipette or graduated cylinder
Toluene	12 mL	Use a 10 mL glass pipette
Hydrochloric acid	1.68 mL	Use a 1 mL glass pipette
C17:1 internal standard (10 mg/mL)	25 μL	Use a glass syringe
**Total**	**120 mL**	–

Use immediately after preparation for Step 10b.

**CRITICAL:** These are critical reagents. Prepare in a large glass bottle and measure reagents using glassware (see notes).

## Step-by-step method details

### Animals and design

This section outlines the number of animals prior to initiation of absorption studies.

1. Perform power analysis before initiating the experiment to determine the minimum number of mice required per group.

***Note:*** In our experience, *n* = 8–10 mice per group is typically sufficient to detect differences in fecal lipids. For example, *n* = 10 mice per group provides 80% power to detect a 20% difference between groups at a significance level of *p* < 0.05, assuming a standard deviation of 15%, based on power calculations performed in R (R Core Team).

2. Maintain mice on the experimental diet until ready to initiate absorption studies.

***Note:*** The exact timing depends on the study design, but for our experiments in Chan *et al*,^1^ the timing can range from a few days to 8**–**12 weeks.

### Feces collection

**Timing: 6–8 days**

This section describes collection of feces to quantify fecal energy loss, fecal fatty acid composition, and fatty acid absorption.

3. Collection of feces for fecal excretion measurements (TIMING: 5–7 days).
  ***Note:*** Physiological adaptations may occur during the start of any experimental intervention. To avoid capturing these early adaptive responses, we recommend beginning these absorption measurements ≥ 7 days after experiment onset.
  ***Note:*** The period of fecal collection can be determined by the investigator. In our studies, fecal collection over 7 days yields ∼2 g feces per mouse, which is sufficient for replicate measurements for our bomb calorimetry analyses.
  a. At the start of the fecal collection period, transfer mice to individual standard cages containing white paper bedding (or a similar non-chip bedding) to facilitate visualization and collection of fecal pellets (Figure 2A).
    ***Note:*** Individual housing allows accurate measurement of fecal output for each mouse and prevents cross-contamination of feces between animals.
    ***Note:*** Once mice are individually housed, they should not be returned to group housing due to the risk of aggression and social stress upon reintroduction.

**Figure 2:**
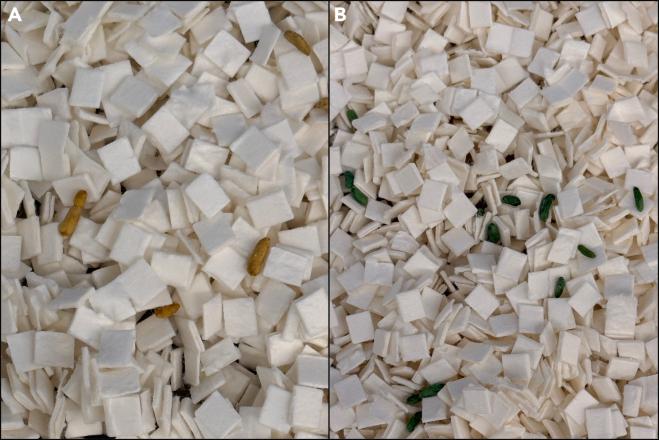
Collection of fecal pellets on white paper bedding (A) Feces from a mouse fed Western diet. (B) Feces from a mouse fed sucrose polybehenate-containing Western diet.
  b. During the fecal collection period, use tweezers to collect all fecal pellets into a labeled conical tube from each cage.
    ***Note:*** If food crumbles are present in the cage, gently brush food particles off fecal pellets during collection.
  c. Record the total fecal mass (g) obtained over the collection period.
  d. Store the feces at −20°C until analysis for fecal energy content by oxygen bomb calorimetry.
    i. Samples can be stored for up to 6 months.
    ii. These fecal samples will be referred to as “non-SPB feces” throughout the text and used to measure fecal energy excretion and fatty acid composition.
4. Collection of feces for fatty acid absorption measurements (TIMING: 1 day).
  a. Following completion of feces collection in Step 3, replace the experimental diet with the blue SPB-containing diet.
  b. After 24 h of SPB diet feeding, collect 5**–**7 pellets of blue-colored feces into a labeled Eppendorf tube for each mouse (Figure 2B).
    ***Note:*** The blue color in the feces confirms excretion of the SPB-containing diet. Because the fatty acid composition of SPB will be determined, complete feces collection and direct measurement of food intake over the 24 h are not required.
  c. After feces collection, replace the blue SPB-containing diet with the original experimental diet.
  d. Store the feces at −20°C until analysis for fatty acid absorption by GC-MS.
    i. These fecal samples will be referred to as “SPB feces” throughout the text and used to measure fatty acid absorption.
      ***Note:*** Feces collection can be performed weekly to capture changes in intestinal fat absorption over time. More frequent collections may be performed if acute interventions are introduced.

### Determination of fecal energy excretion

**Timing: 4–8 h (depending on number of samples)**

This section describes measurement of fecal energy content by oxygen bomb calorimetry.

5. To prepare a single run for the oxygen bomb calorimeter, weigh out 0.8–1 g of non-SPB feces from Step 3 (TIMING: 10**–**20 min, depending on number of samples).

***Note:*** We recommend technical replicates of 2 to 3 runs per sample, depending on the amount of feces available.

***Note:*** The fecal pellet weight range required for each run was optimized and suitable for feces from wild-type mice fed standard rodent diet, wild-type mice fed Western diet, and mice with severe dietary fat malabsorption.

6. To obtain the energy density (heat of combustion; kcal/g) for each sample, operate the oxygen bomb calorimeter according to manufacturer instructions. Average the energy density for technical replicates, if applicable (TIMING: 4-8 h, depending on number of samples).

**Figure 3:**
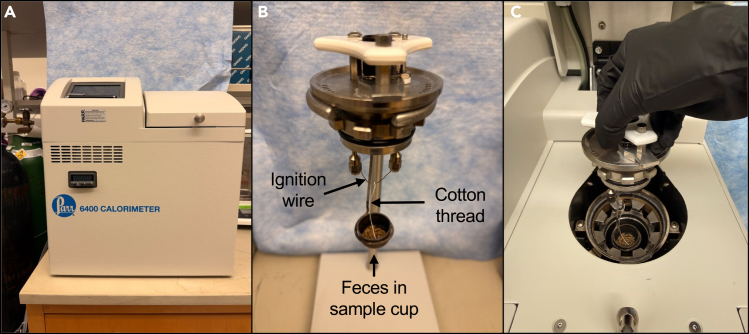
Steps to use oxygen bomb calorimeter for fecal energy analysis (A) Oxygen bomb calorimeter (6400 isoperibol calorimeter with an 1138 oxygen bomb, Parr Instruments Company). (B) Bomb head loaded with feces sample. For a single run, place 0.8–1 g of non-SPB feces in the sample cup. A single piece of cotton thread is looped over the ignition wire, then doubled on itself, twisting to form a single strand to touch the sample, according to manufacturer’s instructions for this calorimeter. (C) Securing the loaded bomb head onto the bomb cylinder according to manufacturer’s instructions.

***Note:*** We used a 6400 isoperibol calorimeter with an 1138 oxygen bomb (Parr Instruments Company, Moline, IL) (Figure 3). On our calorimeter, each sample takes nine min including sample preparation time. For 20 mice with duplicate measurements, the total time is 6 h.

7. For each sample, multiply the cumulative fecal mass (g) with energy density (kcal/g) to determine total fecal energy loss (kcals) over the specified time period (TIMING: 10 min).

***Note:*** For example, a cumulative fecal mass of 10 g multiplied by energy density of 4.7 kcal/g will result in fecal energy loss of 47 kcals.

### Quantification of fatty acid absorption

**Timing: 3–6 days (depending on number of samples)**

This section describes measurement of fecal fatty acid composition and fatty acid absorption by GC-MS.

**Figure 4:**
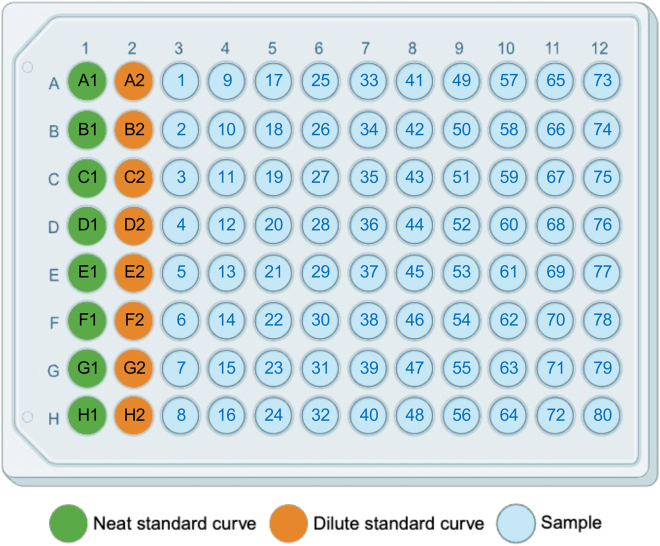
96-well plate layout for standards and fecal for gas chromatography-mass spectrometry analysis
    iii. Include additional plate positions containing the SPB compound and the SPB-containing diet.
      **CRITICAL:** Calculation of fatty acid absorption using the Jandacek method^2^ requires analyses of fecal samples, the SPB compound, and the SPB-containing diet. The SPB compound is analyzed to account for tracer impurities, and the diet is analyzed to allow calculation of percent absorption.
  b. Homogenize the feces in liquid nitrogen using mortar and pestle (Figure 5).
    ***Note:*** In our hands, it takes ∼2 min per sample, or 3 h for a full plate of 80 samples.

8. Fecal sample preparation for GC-MS (TIMING: 4-8 h, depending on number of samples).
  ***Note:*** While we prepare extracts in a specialized plate format, all steps can be adapted to screw-top glass tubes and glass autosampler vials, depending on individual extraction and autosampler setups for mass spectrometer analysis.
  a. Prepare a GC-MS sample list and corresponding 96-well plate template.
    i. Reserve the first two columns for standards, which includes blanks.
    ii. Assign the remaining wells to non-SPB feces from Step 3 and SPB feces from Step 4 (Figure 4).

**Figure 5:**
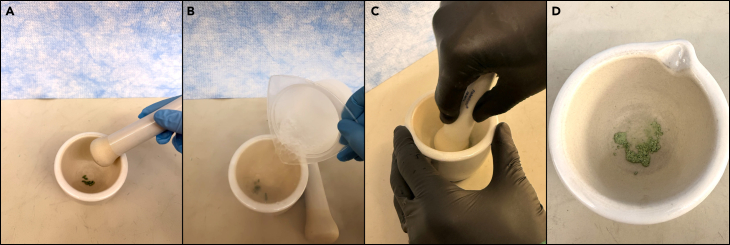
Steps to powder fecal samples using mortar and pestle (A) Fecal pellets in mortar. (B) Liquid nitrogen added to feces in mortar. (C) Feces are powdered in liquid nitrogen using mortar and pestle. (D) Powdered feces.
  c. Weigh 5 mg powdered feces directly into 2.5 mL glass vial inserts. Record the exact mass for each sample.
    ***Note:*** In our hands, it takes ∼2 min per sample, or 3 h for a full plate of 80 samples.
    ***Note:*** Larger amounts of feces may be used provided that all reaction volumes are scaled proportionally by sample weight. In mice fed a high-fat diet (e.g., Western diet), fecal lipid content is high, so this protocol is optimized to use the lowest fecal mass that can be weighed accurately.
    ***Note:*** Feces are powdered to increase surface area and improve the efficiency of the lipid extraction using a fine balance.
  d. Position each fecal sample tube according to the pre-planned 96-well plate template in Step 8a.
  e. Weigh 3 mg of SPB from the compound reserved in Step 2 of “Preparation of diet containing sucrose polybehenate (SPB)” and load into a 2.5 mL glass vial insert according to the predefined plate map.
    ***Note:*** There may be batch-to-batch variation in commercially sourced SPB. Therefore, we recommend confirming SPB purity by determining the exact fatty acid composition of the SPB used for the study. In our batch, SPB consists predominantly of behenate (C22:0), with minor contributions from eicosanoate (C20:0), stearate (C18:0), and other fatty acids (Table 1).
    **Table 1:** Fatty acid composition of sucrose polybehenate, related to Step 8

Name	Chain	% Composition
Myristate	C14:0	0.05
Palmitate	C16:0	0.7
Palmitoleate	C16:1	0.005
Stearate	C18:0	2.3
Oleate	C18:1	0.05
Linoleate	C18:2	0.01
Eicosanoate	C20:0	7.7
Eicosenoate	C20:1	0.02
Behenate	C22:0	87.1
Lignocerate	C24:0	2.6
9. Preparation of standard mixtures and calibration curves (TIMING: 1 hour).
  **CRITICAL:** Perform the following steps in a chemical fume hood due to the use of hexane. Hexane is a carcinogen and inhalation exposure may cause reproductive harm.
  **CRITICAL:** Plastics should not be used for any step involving solvents to minimize contamination from the plastic that can impact fatty acid quantification.^7^
  a. Prepare 3 solvent-rinsed and dried glass flasks of hexane to wash glass syringes in between every use.
  b. Prepare 10 mg/mL Standard Mix Stock in a screw-top glass tube by dissolving 100 mg of GLC96C in 10 mL hexane. Vortex to mix.
    i. This standard mix will be used to generate the neat standard curve.
      ***Note:*** After every use, store the Standard Mix Stock in a parafilm-wrapped screw-top glass vial at −20°C. Before reuse, allow the solution to equilibrate to 25°C and vortex to suspend. Mark the hexane level on the vial with a permanent marker after each use to monitor evaporation. Prepare a fresh stock if evaporation is detected.
  c. Prepare 1:20 Standard Mix in a 1.5 mL screw-top amber vial by mixing 20 μL of Standard Mix Stock in 380 μL hexane using appropriate glass syringes. Vortex to mix.
    i. This standard mix will be used to generate the dilute standard curve.
  d. Wash glass syringes with hexane, as prepared in Step 9a.
  e. For the neat standard curve, add the following volumes of Standard Mix Stock into the dedicated 2.5 mL glass vial inserts (first column) using glass syringes:
    i. A1: 0 μL.
    ii. B1: 1 μL.
    iii. C1: 5 μL.
    iv. D1: 10 μL.
    v. E1: 20 μL.
    vi. F1: 40 μL.
    vii. G1: 80 μL.
    viii. H1: 160 μL.
  f. For the dilute standard curve, add the following volumes of 1:20 Standard Mix into the dedicated 2.5 mL glass vial inserts (second column) using glass syringes:
    i. A2: 0 μL.
    ii. B2: 1 μL.
    iii. C2: 5 μL.
    iv. D2: 10 μL.
    v. E2: 20 μL.
    vi. F2: 40 μL.
    vii. G2: 80 μL.
    viii. H2: 160 μL.
  g. Cap the glass vials with the solvent-safe silicone liner.
10. Preparation of fatty acid methyl esters by acid methanolysis (TIMING: 2 days).
  a. Prepare 10 mg/mL trinonadecanoin (C17:1) Internal Standard Stock in screw-top glass tube by dissolving 100 mg of C17:1 in 10 mL hexane in a fume hood. Vortex to mix.
    i. This stock solution can be stored for up to 6 months.
      **CRITICAL:** C17:1 is used as an internal standard for GC-MS quantification because it is present in biological samples at only trace levels, minimizing interference with endogenous fatty acid measurements while correcting for any variability in extraction or derivatization between runs. Although other odd-chain fatty acids, such as C15:0 and C19:0, are also used as internal standards for other applications, these fatty acids are present in Western diet (Research Diets D12079B) and thus should not be used as standards for this application. Check diets for potential contaminating fatty acids before selecting internal standards.
  b. Prepare acid methanolysis mix in a large glass bottle immediately before use.^8^^,^^9^ See materials and equipment for reagents and recipe.
    ***Note:*** This yields a total volume of 120 mL, providing > 50% excess to account for pipetting losses and dead volume associated with the reservoir bottle and automated liquid handling. For manual pipetting, the total volume may be reduced, but reagent ratios should be maintained for methanolysis.
  c. Add 720 μL of acid methanolysis mix into the glass vials containing the standards and fecal samples using a glass pipette or automated liquid handler.
    ***Note:*** Detailed setup and operation of the liquid handler have been described previously in a step-by-step protocol.^10^
  d. Cap the glass vials with a solvent-safe silicone liner.
  e. Incubate the plate containing sealed glass vials for 10**–**16 h at 45°C.
    ***Note:*** This step is acid methanolysis, which converts free fatty acids and fatty acyl chains from complex lipids, such as triglycerides, diglycerides, cholesterol esters, and phospholipids, into fatty acid methyl esters (FAMEs). In feces, most lipids are present as free fatty acids and thus comprise the majority of fecal FAMEs (Figure 6). For concerns about incomplete methanolysis, refer to troubleshooting – problem 2.
    **CRITICAL:** Perform the following steps in a chemical fume hood to prevent inhalation or wear an appropriately fitted half-mask respirator in a room with adequate ventilation.

**Figure 6:**
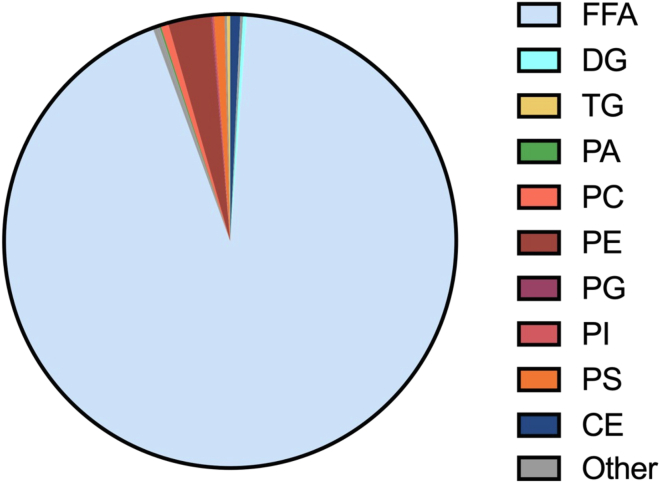
Distribution of lipid classes in feces by lipidomic analysis Feces were collected from wild-type mice after two weeks of Western diet feeding. FFA, free fatty acid; DG, diacylglyceride; TG, triglyceride; PA, phosphatidic acid; PC, phosphatidylcholine; PE, phosphatidylethanolamine; PG, phosphatidylglycerol; PI, phosphatidylinositol; PS, phosphatidylserine; CE, cholesterol ester.
  f. After incubation, remove the plate from the incubator and uncap the glass vials.
  g. Dispense 880 μL of hexane into every glass vial followed by 880 μL 0.04 M sodium chloride.
    ***Note:*** The salt precipitates residual proteins and FAMEs partition into the upper hexane layer.
  h. Seal tubes using a solvent-safe replacement liner and aluminum cover, then invert plate 5-7 times to mix.
  i. Balance plate in centrifuge with a swing-bucket plate rotor.
  j. Centrifuge at 320 × *g* for 5 min.
  k. During the spin, prepare a new plate with empty 1.5 mL glass vial inserts in the same configuration as the 96-well plate template in Step 8a.
  l. After the spin, transfer the upper hexane layer containing the lipid extract to the corresponding 1.5 mL glass vial inserts using a Pasteur pipette or liquid handler.
    ***Note:*** If a centrifuge suitable for tall glass inserts is not available, samples can be placed on a flat surface inside a fume hood for 1 to 2 h to allow passive phase separation. The hexane layer will gradually rise to the top. Using a Pasteur pipette, transfer 2/3 to 3/4 of the upper hexane layer containing the lipid extract to the corresponding 1.5 mL glass vial inserts.
  m. Apply pierceable cap mat to seal the plate. Samples are now ready for the GC-MS run.
    ***Note:*** Pre-warm the pierceable cap mat at 45°C to facilitate sealing.
  n. Inject 1 μL of sample for analysis.
    ***Note:*** This amount of extract from ∼5 mg of feces has been optimized for our GC-MS system. Fecal lipid extracts are highly concentrated and may require additional dilution to prevent overloading of the GC column and ensure analyte abundance falls within the linear dynamic range of the detector. This should be assessed for individual experiments. Perform a pilot run with a small number of samples to determine optimal dilution, and prepare additional dilutions as needed.
    ***Note:*** Solvent evaporation can introduce minor variability. All samples are normalized to the C17:1 internal standard added during extraction, which corrects for variability due to pipetting and post-extraction evaporation.
    ***Note:*** Samples will evaporate over 48**–**72 h and should be run on the GC-MS instrument as soon as possible. If the run must be delayed, store samples at 4°C and replenish hexane as needed to maintain consistent volumes.
11. GC-MS analysis (TIMING: 1**–**3 days, depending on number of samples and GC-MS instrument).
  a. Complete GC-MS analysis optimized for the system available.

***Note:*** GC-MS analysis can be performed using a variety of instruments and configurations. This protocol was optimized for use with an Agilent 7890B/5977A system (Agilent Technologies, Santa Clara, CA) with a DB-WAX UI column (Agilent Technologies, 122-7032 UI) installed with a midpoint backflush configuration. Additional details regarding GC-MS configuration and method parameters have been published elsewhere.^10^

***Note:*** FAMEs were identified based on retention time and ion m/z (Table 2) and quantified by comparison to calibration curves generated from standard mixtures, with normalization to the C17:1 internal standard. Retention times are provided as a reference for our GC-MS system and may vary depending on instrument configuration.

***Note:*** Peak integration and calculation of areas under the curve (AUC) for all standards and samples are performed using MassHunter Quantitative Analysis software (Agilent Technologies).

***Note:*** The extracted samples described above will predominately contain fatty acids and various sterols found in the fecal material. These sterols will have very poor mobility on most columns suitable for FAMEs. It will be necessary to backflush these sterols to prevent accumulation on the column, which would affect subsequent samples. This is done within the method for instruments which can support a backflush, or a method with sufficient runtime extension to clear sterols from the column may be used.

12. Calculation of fecal fatty acid composition (TIMING: 1 hour).
  a. Use the AUCs from standards to generate a standard curve. Then, calculate FAMEs as either absolute amount (nmol) or concentration (nmol/mg of sample) for all fecal samples.
    ***Note:*** A spreadsheet template is provided to calculate the amount and concentration of fecal samples (Document S1).
    ***Note:*** Only non-SPB feces are used to measure total fecal fatty acid excretion and fecal fatty acid composition. SPB is nonabsorbable and passes undigested into the feces. As a result, behenic acid (C22:0) and the other fatty acids from SPB can displace other endogenous dietary fatty acids and comprise a substantial fraction of total fecal fatty acids, leading to inaccurate quantification.
  b. To calculate total fatty acid excretion, multiply the total fecal output collected over the defined collection period (g) in Step 3c by the measured fatty acid concentration (nmol/mg) of each non-SPB fecal sample.
    ***Note:*** In the same spreadsheet as above, a template is provided to calculate fecal fatty acid composition (Document S1).
    ***Note:*** If samples fall outside the linear curve, refer to troubleshooting – problem 3.
13. Calculation of fatty acid absorption (TIMING: 2 h).
  a. From the FAMEs calculated in Step 12a, calculate the relative abundance of each FAME as a percentage of total FAMEs for SPB fecal samples only.
  b. Use the equation below to calculate the absorption of total and individual fatty acids:

$$\frac{\frac{F_{d}}{B_{d}}-\left[\frac{F_{f}-\left(B_{f}\times F_{SPB}\right)}{B_{f}}\right]}{\frac{F_{d}}{B_{d}}}$$

**Table 2:** Reference m/z ions for fatty acid methyl esters, Related to Step 11

Name	Chain	Reference ion (m/z)	Retention time (min)
Methyl myristate	C14:0	242	6.3
Methyl palmitate	C16:0	270	8.0
Methyl palmitoleate	C16:1	268	8.2
Methyl heptadecanoate	C17:0	284	8.9
Methyl heptadecenoate	C17:1	282	9.2
Methyl stearate	C18:0	298	9.9
Methyl oleate	C18:1	296	10.1
Methyl linoleate	C18:2	294	10.5
Methyl alpha linolenate	C18:3	292	11.1
Methyl eicosanoate	C20:0	326	11.8
Methyl 11-eicosenoate	C20:1	324	11.9
Methyl 11-14-eicosadienoate	C20:2	322	12.2
Methyl homogamma linolenate	C20:3	320	12.4
Methyl arachidonate	C20:4	318	12.6
Methyl behenate	C22:0	354	13.2
Methyl lignocerate	C24:0	382	14.9

F_d_ = % of total fatty acid minus behenic acid, or the individual fatty acid, in diet,

B_d_ = % of behenic acid in diet,

F_f_ = % of total fatty acid minus behenic acid, or individual fatty acid, in feces,

B_f_ = % of behenic acid in feces.

F_SPB_ = % of individual fatty acid in SPB.

***Note:*** Although SPB consists predominantly of behenic acid (C22:0), it contains minor amounts of other fatty acid species. These minor components can contribute to measured fecal fatty acid pools and confound quantification of endogenous dietary fatty acids. Thus, a correction factor is incorporated into the calculations to correct for the contribution of these minor fatty acid species.

***Note:*** In the same spreadsheet as above, a template is provided to calculate total and individual fatty acid absorption (Document S1).

## Expected outcomes

**Figure 7:**
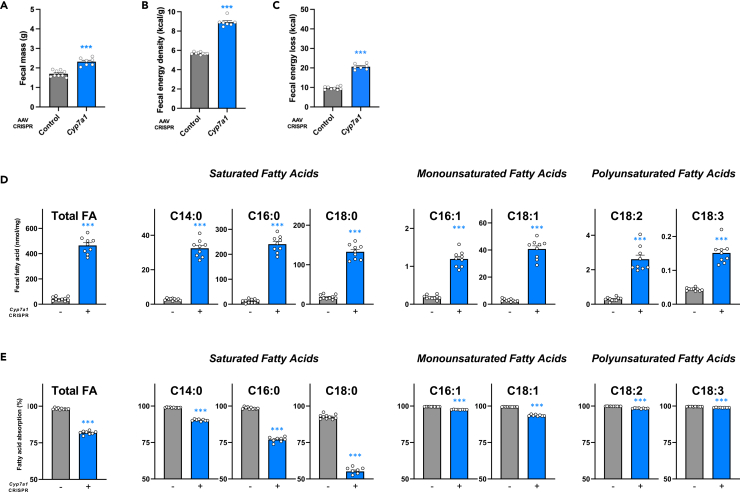
Reducing bile acid pool size with loss of CYP7A1 selectively promotes fecal energy excretion and decreases fatty acid absorption Adapted from Chan *et al.*^1^ (A–E) Total fecal mass (A), fecal energy density (B), total energy loss (C), fecal fatty acid concentration (D), and fatty acid absorption (E) during the final week in control and *Cyp7a1* AAV-CRISPR mice after eight weeks of Western diet feeding. *n* = 7–10 mice per group. Data are represented as mean ± SEM. ∗∗∗*p* < 0.001, by two-sided t-tests.

Intestinal absorption of dietary fatty acids is highly efficient and generally exceeds 90%.^11^ The example data illustrates the range of results expected in adult mice fed a Western diet for eight weeks under *ad libitum* feeding conditions (Figure 7). In control mice, polyunsaturated fatty acids are preferentially absorbed over saturated fatty acids, reflecting differential fatty acid absorption. For comparison, data from mice lacking hepatic *Cyp7a1* (disrupted using AAV-CRISPR), where the bile acid pool size is reduced, are also shown, as previously reported.^1^ In these mice, total intestinal fatty acid absorption is reduced by approximately 30% and accompanied by increased fecal energy and fat loss. Notably, reduced bile acid levels cause a decrease in the absorption of saturated fatty acids, which is more substantial compared with unsaturated fatty acids, accentuating the natural selectivity of bile acids in fatty acid absorption. Importantly, these data also highlight the sensitivity of the SPB assay to detect biologically meaningful differences in intestinal absorption of both saturated and unsaturated fatty acids.

## Limitations

The following points should be considered when applying this protocol. As this SPB assay was optimized in mice fed a Western diet, the SPB dosage may require adjustment when used with other experimental diets or animal strains. Although this approach provides a sensitive measure of net intestinal fatty acid absorption, it does not resolve regional differences in intestinal lipid uptake, standardize the amount of lipid administered, or capture post-absorptive lipid handling, such as chylomicron secretion or peripheral tissue uptake. Finally, lipid extraction is performed using acid methanolysis, which converts free fatty acids and fatty acyl chains from complex lipids, including triglycerides, diacylglycerides, phospholipids, and cholesterol esters, into FAMEs for GC-MS analysis. As a result, the measured FAMEs reflect total fecal fatty acids rather than exclusively free fatty acids. Because free fatty acids are the predominant lipid species in feces (Figure 6), the FAMEs procedure is unlikely to affect interpretation of changes in absorption.

## Troubleshooting

### Problem 1

Behenic acid (C22:0) is present at negligible amounts in most dietary fat sources used in rodent diets (see “Preparation of diet containing sucrose polybehenate (SPB)” section). Because this protocol calculates fat absorption from the ratio of behenic acid to other fatty acids in diet and feces, any endogenous C22:0 in the diet will inflate the dietary C22:0 amount beyond what is contributed by SPB alone, causing the calculated fatty acid absorption to appear falsely low.

### Potential solution

• If the fatty acid absorption is significantly lower than expected, confirm that the experimental diet does not contain appreciable amounts of endogenous C22:0.
• If endogenous C22:0 is present in the diet and cannot be reduced with another fat source, consider increasing the percentage of SPB incorporated into the diet to minimize the background noise, but not above 3% of final composition. Alternatively, the amount of endogenous C22:0 in the experimental diet can be subtracted from the total dietary C22:0 value in the SPB-containing diet.

### Problem 2

Incomplete acid methanolysis will result in poor conversion of lipids to FAMEs, meaning a proportion of the sample remains as unesterified fatty acids or other lipid species rather than the corresponding methyl esters (see Step 10). Because downstream calculations of fatty acid absorption are based on FAME quantification, incomplete conversion across fatty acid species can complicate the calculation of true fatty acid content.

### Potential solution

• Incomplete acid methanolysis can be verified by running lipid standards of known fatty acid composition alongside samples. Standards should produce peaks at the expected m/z for FAMEs at signal intensities consistent with the amount of standard used. If peaks corresponding to non-methylated lipid species are observed, or if FAME signal intensity is lower than expected for the standard amount, incomplete conversion should be suspected.
• Ensure that acid methanolysis mixture is prepared freshly, within an hour of addition to samples for each conversion, as reagent degradation can reduce conversion efficiency.
• Verify that reaction conditions, including temperature and incubation time, are strictly maintained according to the protocol, as deviations can result in incomplete methyl esterification.

### Problem 3

Calculation of the absolute amount (nmol) or concentration (nmol/mg of sample) of fecal fatty acids is based on calibration curves generated from standard mixtures and GC-MS configurations (see Step 12). Sample values should fall within the linear range of the calibration curve. If values fall outside this range, calculated values may be inaccurate.

### Potential solution

• If FAME levels fall below the linear range, include a more dilute standard curve for the GC-MS run. For example, prepare a 1:100 or 1:200 dilution of the neat standard mixture and set up in column 3 of the 96-well plate template.
• If FAME levels exceed the linear range, dilute fecal samples prior to acid methanolysis. Because accurately weighing fecal samples below 5 mg can be challenging, we recommend preparing a fecal suspension by homogenizing 5 mg of feces in 100 μL of 1x PBS in a homogenizer tube pre-loaded with 2.8 mm ceramic beads (OMNI #19-628) using a homogenizer (e.g., Bead Ruptor Elite, OMNI International). Aliquot 10 μL of the suspension into the glass insert vial to achieve a 1:10 dilution prior to lipid extraction.

## Resource availability

### Lead contact

Requests for further information and resources should be directed to and will be fulfilled by the lead contact, Thomas Q. de Aguiar Vallim (tvallim@mednet.ucla.edu).

### Technical contact

Questions about the technical specifics of performing the protocol should be directed to the technical contact, Alvin P. Chan (alvinchan@mednet.ucla.edu).

### Materials availability

This study did not generate new unique reagents.

### Data and code availability

This study did not generate code or analyze datasets.

## Acknowledgments

This work is primarily supported by R01DK138340 from the 10.13039/100000002National Institutes of Health to T.Q.d.A.V. T.Q.d.A.V. and E.J.T. are also supported by R01HL174008, R01HL163908, and R01DK128952. E.J.T. is supported by the 10.13039/100000968American Heart Association (23EIA1037961). A.P.C. is supported by the Mentored Clinical Scientist Development Award from the 10.13039/100000062National Institute of Diabetes and Digestive and Kidney Diseases of the National Institutes of Health (1K08DK146180-01), Child Health Research Career Development Award from the 10.13039/100000071National Institute of Child Health and Human Development of the National Institutes of Health (1K12HD111040), the UCLA Office of Physician Scientist Career Development Bridge Funding Grant, 10.13039/100005595UCLA Children’s Discovery and Innovation Institute Seed Grant (CDI-SEED-010124), Gastroenterology Training Grant from the 10.13039/100000062National Institute of Diabetes and Digestive and Kidney Diseases of the National Institutes of Health (T32DK007180), and the UCLA Children’s Discovery and Innovation Institute Fellow Support Award (CDI-FRSA-07012021). K.E.J. is supported by the American Heart Association Career Development Award (26CDA1608262), Iris Cantor Women’s Health Center pilot grant, and UCLA SCORE on Cardiometabolic Health and Disease pilot grant (U54HL170326). A.P.C., K.E.J., and T.Q.d.A.V. are supported by pilot and core voucher grants from the UCLA 10.13039/100010554Clinical and Translational Science Institute (UL1TR001881). R.W.L. is supported by the UCLA Goodman-Luskin Microbiome Center Seed Fellowship and the UCLA Graduate Programs in Bioscience Asrican Sophie & Jack Award. GC-MS analyses were performed with support from the UCLA Lipidomics Core Laboratory. Diet preparation and feces collection were performed by Andrew Lau and Owen K. Traina. Figure schematics were generated using www.Biorender.com and FigureLabs (figurelabs.ai).

## Author contributions

Protocol optimization and writing were carried out by A.P.C., K.E.J., and R.W.L. and overseen by E.J.T. and T.Q.d.A.V. Editing was performed by B.L.C., K.J.W., E.J.T., and T.Q.d.A.V. Figures were generated by R.W.L., A.P.C., and K.E.J.

## Declaration of interests

The authors declare no competing interests.

## Declaration of generative AI and AI-assisted technologies in the writing process

During the preparation of this work, the authors used ChatGPT (OpenAI) in order to refine language and improve clarity in the text. After using this tool, the authors reviewed and edited the content as needed and take full responsibility for the final version of the publication.
